# Ten years follow-up of the largest oral Chagas disease outbreak: Cardiological prospective cohort study

**DOI:** 10.1371/journal.pntd.0011643

**Published:** 2023-10-06

**Authors:** Raiza Ruiz-Guevara, Belkisyolé Alarcón de Noya, Iván Mendoza, Cielo Rojas, Iván Machado, Zoraida Díaz-Bello, Arturo Muñoz-Calderón, Julio Castro, Oscar Noya

**Affiliations:** 1 Cátedra de Parasitología, Escuela ¨Luís Razetti” Facultad de Medicina, Universidad Central de Venezuela, Caracas, Venezuela; 2 Instituto de Medicina Tropical, Facultad de Medicina, Universidad Central de Venezuela, Caracas, Venezuela; 3 Departmento de Cardiología, Sección de Enfermedades Cardíacas Congénitas, Hospital Universitario de Caracas, Universidad Central de Venezuela, Caracas, Venezuela; 4 Centro para Estudios Sobre Malaria, Instituto de Altos Estudios “Dr. Arnoldo Gabaldón”, Ministerio del Poder Popular para la Salud (MPPS), Caracas, Venezuela; Federal University of Minas Gerais, BRAZIL

## Abstract

**Background:**

Chagas disease (ChD) is the most important endemy in Latin America. Some patients, develop chronic Chagasic cardiopathy (CCC) years after the acute phase. It is unknown if patients infected by the oral route have higher risk of developing early CCC.

**Methods and findings:**

A prospective cohort study was conducted to assess morbidity and mortality during 10 years observation in 106 people simultaneously infected and treated in the largest known orally transmitted ChD outbreak in 2007. A preschooler died during the acute phase, but thereafter was no mortality associated to ChD. All acute phase findings improved in the first-year post-treatment. Each person was evaluated 8.7 times clinically, 6.4 by electrocardiogram (ECG)/Holter, and 1.7 by echocardiogram. Based on prevalence, the number of people who had any abnormalities (excluding repolarization abnormalities and atrial tachycardia which decreased) was higher than 2007, since they were found at least once between 2008–2017. However, when we evaluated incidence, except for clinical bradycardia and dizziness, it was observed that the number of new cases of all clinical and ECG findings decreased at the end of the follow-up. Between 2008–2017 there was not incidence of low voltage complex, 2^nd^ degree AV block, long QT interval, left bundle branch block or left ventricular dysfunction that allowed the diagnosis of CCC. Total improvement prevailed over the persistence of all clinical and ECG/Holter findings, except for *sinus bradycardia*. *Incomplete* right bundle branch block, sinus bradycardia and/or T-wave inversion were diagnosed persistently in 9 children. The second treatment did not have significant influence on the incidence of clinical or ECG/Holter findings.

**Conclusions:**

At the end of the 10-year follow-up, there were not clinical or ECG/Holter criteria for classifying patients with CCC. The incidence of arrhythmias and repolarization abnormalities decreased. However, special attention should be paid on findings that not revert as sinus bradycardia, or those diagnosed persistently in all ECG as sinus bradycardia, incomplete right bundle branch block or T-wave inversion. Early diagnosis and treatment may have contributed to the rapid improvement of these patients. In ChD follow-up studies prevalence overestimates the real dimension of abnormalities, the incidence looks as a better indicator.

## Author summary

Chagas disease (ChD) produced by *Trypanosoma cruzi*, usually, infect humans through the contact with the feces of blood-sucking triatomines, but microepidemics of oral transmission have been described. We ignore if patients who were infected by the oral route have a higher risk of developing early chronic Chagasic cardiopathy (CCC). In this work, a 10 years prospective cohort study was carried out to evaluate 106 people who were infected and treated simultaneously during the largest outbreak of orally transmitted ChD described. All clinical, electrocardiographic and ultrasonographic alterations of the acute phase improved almost completely few weeks after specific treatment. However, clinical and electrocardiogram bradycardia were the only non-subjective findings that did not revert during follow-up. Other findings appeared and sometimes persisted which do not necessarily indicate CCC, arising question if these could be early signs of CCC. A 5-years-old preschooler died during the acute phase, but there was no mortality associated with ChD during follow-up. Rapid diagnosis and immediate anti-parasitic treatment could be the reasons for the good evolution of these patients.

## Introduction

Chagas disease (ChD) is one of the highest burden neglected tropical diseases (NTDs) in Latin America and the Caribbean [1,2]. From 25–90 million of persons at risk, there are 8–9 million who are currently infected [3]. ChD mainly affects poor people living in rural communities, who often get infected through triatomines [1]. However, the migration of people from rural to urban areas and the adaptation of wild vectors to the peripheries of large cities as Caracas, have allowed the establishment of enzootic cycles between reduvids such as *Panstrongylus geniculatus* and synanthropic mammals and humans, facilitating the contamination of food by infected triatomines or their feces [4,5,6]. Additionally, human-to-human transmissions (vertical mother–child infection, transfusions, organ transplants, reactivation), have changed the epidemiology and the clinical evolution of ChD in the urban environment [6].

Outbreaks of acute orally transmitted ChD (OChD) have been described since 1968 [7]. OChD usually affects simultaneously more than one person with acute febrile disease without other causes except its linked to a suspected shared meal [8,9]; people are infected with multiples clones of the same genotype of *Trypanosoma cruzi*, and differ only by the dose of the inoculum, age, genetic factors and pre-existing co-morbidities. Generally, the oral outbreak communities occur in endemic areas where previous contact with the parasite cannot be ruled out. The Chacao outbreak occurred at a distance from the contamination site [4,8], so it can be stated that this middle-class school population, was not previously exposed to the parasite.

Throughout 10 years of very close control of Chacao patients with OChD, we have a broad, unique and a valuable overview, representing an important contribution to ChD research in its more than 100-year history in terms of clinical and laboratory evolution and pharmacovigilance of ChD in these orally infected individuals [10–14].

The acute phase of ChD is followed by the indeterminate chronic form. About 50–70% of infected individuals will remain in this condition for the rest of their lives [3,15], and 10–20 years after the acute infection, between 10–30% will present fibrosis scattered throughout the myocardium which results in the chronic Chagasic cardiomyopathy (CCC) [3,15,16]. ECG abnormalities considered to be attributed to CCC have been widely described [17,18]. ChD is a risk factor for presenting ECG abnormalities compared to non-ChD [19], and since the prevalence of ECG alterations in children has been similar to that in adults, it has been suggested earlier onset of cardiac disease [19]. Mortality is generally high among chagasic individuals who develop chronic cardiopathy, mainly when cardiac failure and/or severe arrhythmias occur [20].

We do not know what the natural history of OChD is and if these patients have a greater propensity or speed to develop the chronic phase with heart and/or digestive involvement. In addition, there are few studies evaluating the long-term efficacy of nifurtimox on clinical and ECG evolution in patients with OChD.

In the present work, the findings of the cardiological follow-up after anti-parasitic treatment for 10 years of the largest outbreak of OChD are reported. Its laboratory monitoring with biomarkers of infection for a period of 10 years has been previously published [12]. We will shortly publish the findings on how clinical and laboratory data correlate in this group of patients. The main objective is to evaluate any clinical or ECG/Holter parameter that can help to determine progress toward the development of CCC in patients orally infected.

## Methods

- **Ethical statements**. This project was approved by the Ethics Committee of the Institute of Tropical Medicine "Dr. Félix Pifano" of the Central University of Venezuela, Caracas, Venezuela (CEC-IMT 019/2010—December 10, 2010); all adult patients or guardians of those under 18 years of age has signed and authorize in writing their free and informed consent to participate in this study.
- **Evaluation period.** Clinical, ECG and laboratory control was performed. The information corresponds to the time prior to treatment in 2007 (acute phase) and post-treatment between 2008–2017.
- **Population evaluated**
  - **Inclusion criteria**. The study involved 106 people with acute ChD (100 of the 103 patients diagnosed in December 2007 and 6 identified in confirmatory tests) [21]. The age of the patients is the one in 2007 and it was maintained during all this study. Children were defined as those aged 18 years or less and adults those over; children between 2–5 years old were considered preschool, 6–11 years old schoolchildren, and between 12 and 18 years adolescents [22].
  - **Exclusion criteria**. Patients diagnosed in 2007 without post-treatment evaluations.
- **History and physical examination**. Palpitations, fatigue/tiredness, precordial pain, dizziness, loss of consciousness were investigated. Arterial hypertension or another possible cause of heart disease was recorded. Heart rate below 60 beats per minute (bpm) was considered bradycardia. When the 2008–2014 symptomatology persisted constantly or disappeared definitively in all evaluations, it was registered as persistence or disappearance of the respective clinical finding. When clinical information was inconsistent or not available, it was classified as unknown. A similar criterion was used to define persistence or disappearance of lesions in the ECG/Holter.
- **Electrocardiogram (ECG)/Holter and echocardiogram (ECHO).** In patients with positive serology against *T*. *cruzi*, the criterion for diagnosing the chronic ChD on the conventional 12-lead ECG, was the presence of at least one typical and persistent electrocardiographic abnormalities defined in Nunes et al (2018) [18] as bradyarrhythmias and conduction system alterations, atrial tachyarrhythmias, ventricular tachyarrhythmias, primary ST- and T-wave abnormalities, pathological Q wave, as the most commonly found [18], also described in Marques et al (2013) [23] which is based on the AHA/ACCF/HRS recommendations for the standardization and interpretation of ECGs [24]. In the absence of any of those alterations, the diagnosis of indeterminate chronic Chagas disease was made. Holter and ECHO were achieved in patients with pathological clinical or ECG findings. Complications suggestive of heart failure, aneurysms or thromboembolisms were investigated.
- **Chest-X -ray**. It was especially requested in the periods 2011–2017. The cardiothoracic index (CTR) was performed using always the same criteria in the AP projection [25].
- **Laboratory tests**. Laboratory studies performed before and after treatment have been extensively described [12,21]. The personnel who processed the laboratory samples were unaware of the clinical status of the patients and vice versa.
- **Treatment.** In the pre-treatment evaluation, nifurtimox (NFX) (Lampit; Bayer Laboratories) 8 mg/Kg/day for 90 days was used in 100 persons, six patients used benznidazole (BNZ) (Rochagan; Roche Laboratories) 6 mg/Kg/day for 60 days. days [10,11]. During follow-up, some patients received a second treatment due to laboratory findings from 2008–2017. On this second occasion, the medication used was BNZ.
- **Methodological characterization**. This is a cohort study with intervention (a second antiparasitic treatment) in which patients with acute OChD in December 2007 were followed up for 10 years to compare the incidence of the risk factor (development of CCC).
- **Statistical analysis.** The data was analyzed with the SPSS 25 for Windows program (Verson25.0; Copyright SPSS Inc., 2017). For quantitative variables, it was used the ANOVA one-way test. For the analysis of categorical variables, the Chi-square test (χ^2^) or Fisher’s exact test was applied. To obtain the cumulative incidence, the number of new cases in each period was divided by the number of those evaluated in the same period and who did not have the condition under study in the previous period (For incidence calculation see S1–S3 Data). For the comparison of prevalences and incidences between each clinical or ECG finding from 2007 and the global findings from 2008–2017, the McNemar test for related samples and the determination of the odds ratio (OR) with its respective confidence interval of 95% (95% CI). In all cases, the significance criterion was *p*<0.05.

## Results

### Evaluated population

**Fig 1:**
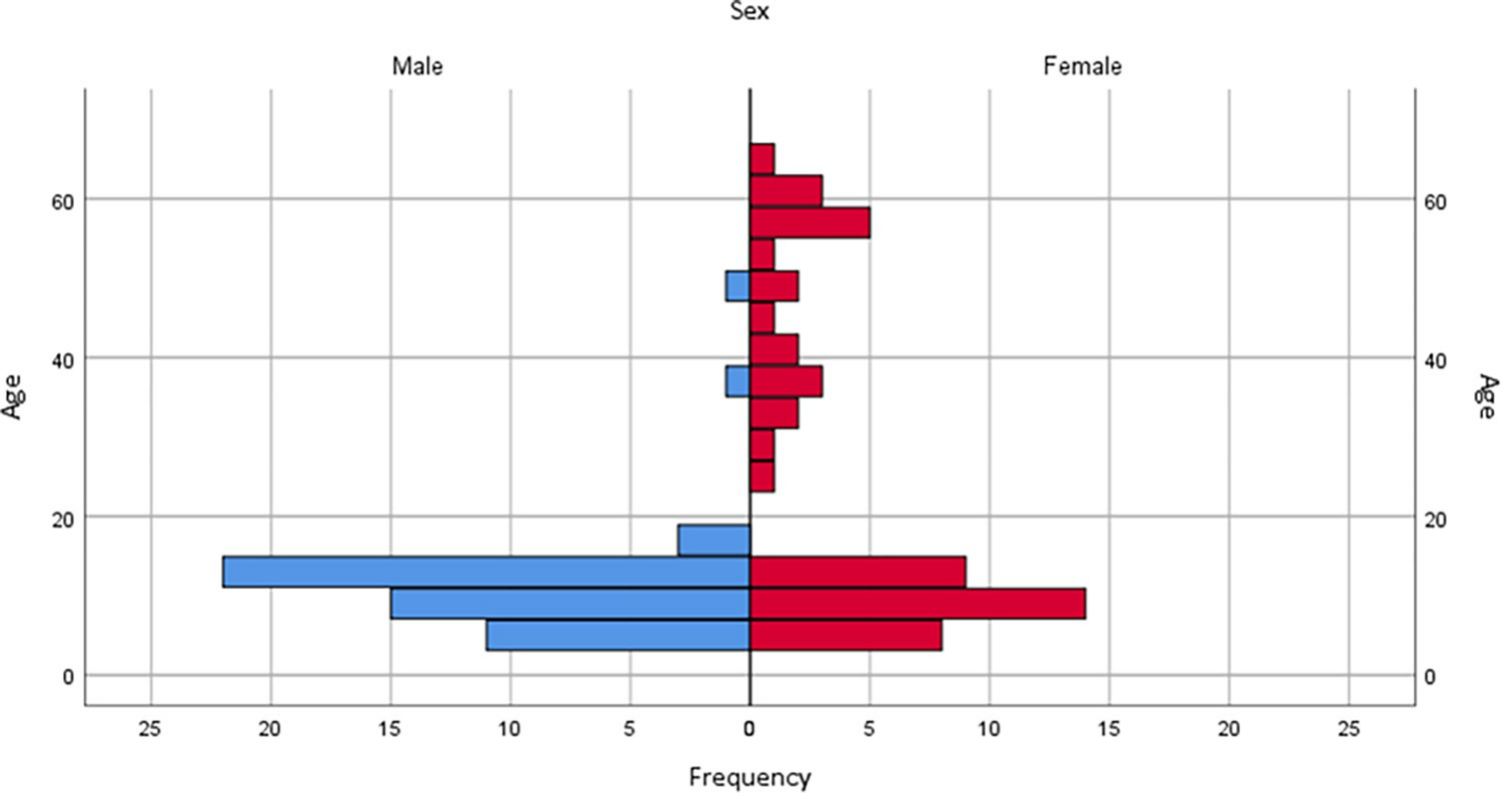
Population pyramid with the age and gender distribution of the 106 patients with Chagas disease acquired by the oral route, 2007–2017, Chacao, Caracas, Venezuela.

Of those evaluated, 53 were men and 53 women with a median age of 11 years; the mean age of the women (24.6 years; SD 20.651) was significantly older than men (10.9 years; SD 7.437) (*p* = 0.000). **Fig 1** represents the age and gender distribution of the entire evaluated population. The study involved 12 preschoolers, 51 schoolchildren, 19 adolescents and 24 adults. **Table 1** shows the number of people evaluated each year or period.

**Table 1:** Absolute number of evaluated patients with oral Chagas disease according to age group before and after the first treatment, Chacao, Caracas, Venezuela 2007–2017.

Evaluated aspects	Age group	Year or period before and after the first treatment					
		Pre-treatment	Post-treatment				
		2007	2008	2009–2011	2012–2014	2015–2017	2008–2017
**Clinic**	Adults	24	24	24	23	23	24
	Children	82	79	82	79	66	82
	Total	106	103	106	102	89	106
**ECG/Holter**	Adults	20	24	24	24	16	24
	Children	66	78	80	69	55	82
	Total	86	102	104	93	71	106
**Echocardiogram**	Adults	7	5	9	16	12	21
	Children	9	6	13	66	2	69
	Total	16	11	22	82	14	90
**Chest-X -ray**	Adults	1	2	1	16	23	23
	Children	4	2	3	39	64	70
	Total	5	4	4	55	87	93

**Fig 2:**
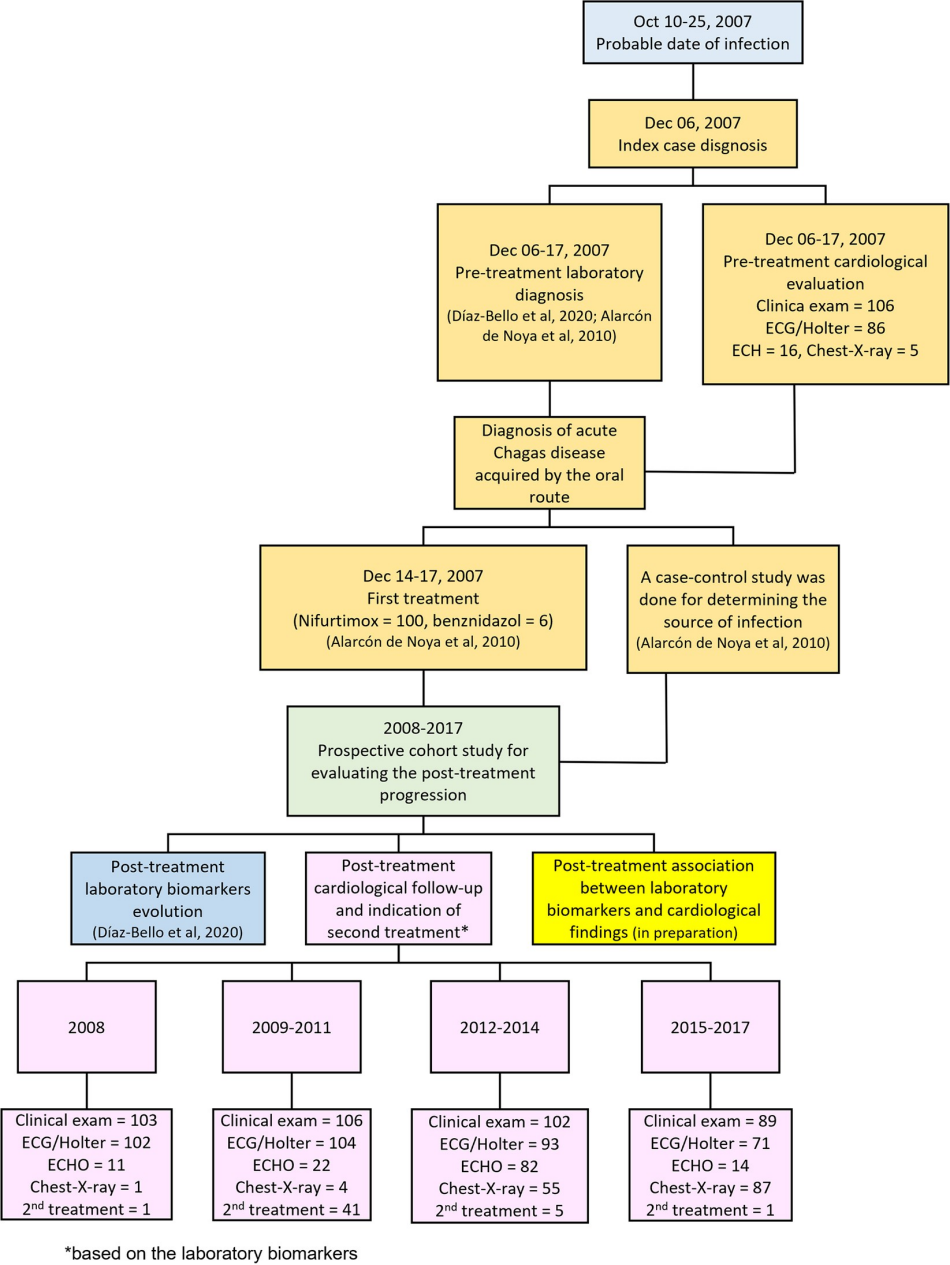
Descriptive flowchart of the complete follow-up of patients with Chagas disease acquired by the oral route, Chacao, Caracas, Venezuela, 2007–2017.

An explicative flowchart of the complete follow-up of the patients with oral Chagas disease of the outbreak occurred in 2007 is shown in **Fig 2**.

### Second treatment

A total of 48 people (9 adults and 39 children, 45.3% of those evaluated) with parasite activity according to laboratory tests carried out during the follow-up [12] received a second anti-parasitic treatment. The times of the second treatment were different. One was treated in 2008, 41 people in the period 2009–2011, five between 2012–2014 and one in 2015–2017 (**Fig 2**).

### Clinical history and physical examination

There were performed 8.7 clinical evaluations (5–11) per person. **Table 2** shows the findings of the interview and physical examination of the patients evaluated between 2007–2017. Of the 11 people with a normal clinical evaluation in 2007, three remained normal in all evaluations and 8 had some type of long-term clinical alterations between 2008–2017. Of 95 people with some pathological finding in 2007, eight were normal during the period 2008–2017 and 87 were not. Of the symptoms reported in 2007, there was constant persistence until 2015 in only 2 people with palpitations and 1 with tiredness/fatigue. On the contrary, there was total disappearance of symptoms in 2007 in 16 persons with palpitations, 7 with tiredness/fatigue, and 5 with precordial pain. An adult died in 2014 for reasons not associated with ChD. Of this group of 106 patients, 94 (88.7%) had various symptoms of the acute phase, and 24 (10 adults and 14 children) required hospitalization; symptoms and ECG alterations of the acute phase disappeared completely in all of them.

**Table 2:** Prevalence of clinical findings and ECG/Holter abnormalities in the follow-up of 106 patients with oral Chagas disease, Chacao, Caracas, Venezuela 2007–2017.

Clinical and ECG/Holter findings		Prevalence Diagnosed/evaluated (%)		Odds ratio and (95% CI)	*p* *
		Pre-treatment 2007	Post-treatment 2008–2017		
**Clinical**	Palpitations	56/106 (52.8%)	64/106 (60.4%)	2.708 (1.214–6.042)	0.268
	Tiredness or fatigue	32/106 (30.2%)	63/106 (59.4%)	3.383 (1.303–8.784)	**0.000**
	Chest pain	15/106 (14.2%)	59/106 (55.7%)	1.43 (1.09–1.87)	**0.001**
	Dizziness	0/106 (0%)	20/106 (18.9%)	…	**0.000**
	Bradycardia	1/106 (0.9%)	37/106 (34.9%)	2.917 (2.238–3.801)	**0.000**
	Fainting? Syncope?	1/106 (0.9%)	1/106 (0.9%)	1.010 (0.991–1.029)	1.000
**ECG/Holter**	Isolated atrial extrasystoles	6/86 (7.0%)	26/106 (24.5%)	7.412 (1.250–43.946)	**0.001**
	Isolated ventricular extrasystoles	4/86 (4.7%)	19/106 (17.9%)	6.308 (3.831–10.386)	**0.000**
	Incomplete right bundle branch block	1/86 (1.2%)	25/106 (23.6%)	4.474 (3.010–6,649)	**0.000**
	Sinus bradycardia	4/86 (4.7%)	25/106 (23.6%)	5.125 (3.302–7.954)	**0.000**
	Sinus tachycardia	15/86 (17.4%)	18/106 (17.0%)	4.773 (1.436–16.861)	0.648
	Repolarization abnormalities**	57/86 (66.3%)	22/106 (20.8%)	1.1 (0.93–1.32)	0.260
	Atrial tachycardia	14/86 (16.3%)	9/106 (8.5%)	3.655 (0.763–17.513)	0.210
	Non-sustained ventricular tachycardia	1/86 (1.2%)	1/106 (0.9%)	…	1.000
	Atrial fibrillation	4/86 (4.7%)	1/106 (0.9%)	0.750 (0.426–1.321)	0.250
	Low voltage	2/86 (2.3%)	0/106 (0%)	…	0.500
	1^st^ AV block	6/86 (7.0%)	0/106 (0%)	…	**0.031**
	2^nd^ AV block	2/86 (2.3%)	0/106 (0%)	…	0.500
	Left bundle branch block	6/86 (7.0%)	0/106 (0%)	…	**0.016**
	Long QT interval	0/86 (0%)	0/106 (0%)	…	…
	Some ECG abnormality***	74/86 (86.0%)	0/106 (0%)	…	**0.000**

*p according to the McNemar χ^2^ test for related samples

**T-wave inversion with or without ST elevation in the acute phase. In chronic phase, only T-wave inversion was found

***Related to Chagas disease

The number of people who had any of these findings at least once between 2008–2017 was higher than that registered in 2007, with significant difference between the prevalence of fatigue, chest pain, dizziness and bradycardia during follow-up compared to pre-treatment (**Table 2)**. During the follow up there was a higher risk of presenting tiredness or fatigue and clinical bradycardia, but before treatment there was a greater risk of presenting palpitations according to the OR values (95% CI).

Palpitations before and after treatment, precordial pain in 2007, and fatigue or tiredness between 2008–2017 were reported significantly more by adults than by children. The OR values and 95%CI suggest a greater probability that an adult will report palpitations and precordial pain compared to children (**Table 3**). Regarding the finding of palpitations between men and women, it was significantly more frequent (*p* = 0.017) among women (71.7%) compared to men (49.1%) between 2008–2017, but the OR values ​​ (0.380) and 95% CI (0.170–0.859) do not suggest a causal relationship between gender and palpitations. In this lapse 38 from 53 women and 26 from 53 men presented palpitations.

The heart rate varied between 58–140 bpm in 2007 and between 40–120 in the 2008–2017 period. Heart rate ​​values ≤ 40 were not found. Loss of consciousness was reported by a 7-year-old girl in 2007 and between 2008–2017 by a 61-year-old woman with a history of hypertension.

**Table 3:** Prevalence of significant associations between the pre and post-treatment clinical and ECG/Holter findings in 106 patients with acute Chagas disease acquired by the oral route, Chacao, Caracas, Venezuela, 2007–2017.

Year or period of evaluation		Abnormality found	Prevalence Diagnosed/evaluated (%)		Odds ratio and (95% CI)	*p* *
			Adults	Children		
**ECG/Holter**	**2007** **(Pre-treatment)**	Sinus tachycardia	9/15 (60%)	6/59 (10.2%)	13.250 (3.490–50.304)	*p* = 0.000
		Atrial tachycardia	6/15 (40%)	8/59 (13.6%)	4.250 (1.189–15.191)	*p* = 0.020
		Ventricular tachycardia	1/15 (6.7%)	0/59 (0%)	5.214 (3.256–8.351)	*p* = 0.000
		Atrial fibrillation	3/15 (20%)	1/59 (1.7%)	14.500 (1.387–151.582)	*p* = 0.003
		Low voltage	2/15 (13.3%)	0/59 (0%)	4.667 (3.099–7.028)	*p* = 0.000
		Repolarization abnormalities **	4/15 (26.7%)	51/59 (86.4%)	0.057 (0.015–0.224)	*p* = 0.000
	**2008–2017** **(Post-treatment)**	Isolated atrial extrasystoles	14/24 (58.3%)	12/82 (14.7%)	12.833 (3.563–46.225)	*p* = 0.000
		Isolated ventricular extrasystoles	10/24 (41.7%)	9/82 (11%)	6.173 (1.961–19.428)	*p* = 0.003

*All the signs and symptoms, as well as the ECG/Holter findings were compared. Those not shown in Table 3 did not have statistical significance

**T-wave inversion with or without ST elevation in the acute phase. In chronic phase, only T-wave inversion was found.

### Effect of the second treatment on the post-treatment clinical evaluation

No statistically significant difference was found between the prevalence of palpitations, tiredness or fatigue, precordial pain, dizziness or clinical bradycardia and whether or not they had received a second treatment.

## ECG/Holter

Of the 106 patients, we have 86 pre-treatment ECG/Holter information, of which 12 were normal and 74 abnormal. Of the 12 normal patients, 10 had one or more alterations after the first treatment and 2 of the 12 remained normal at least until their last evaluation in 2013. Of the 74 abnormal ECG/Holter in the acute phase, 20 were reported normal in all evaluations, and the remaining 54 presented one or several alterations between 2008–2017. After the first treatment, 36 of the 74 (48.6%) abnormal ECGs had normalized. All 86 patients underwent at least one ECG/Holter evaluation between 2008–2017, but, another 20 persons without pre-treatment ECG/Holter, performed ECG/Holter between 2008–2017. **Table 2** shows the ECG/Holter findings performed between 2007–2017. From the ECG/Holter findings of 2007, total disappearance of isolated atrial extrasystoles was found in 2 people, sinus tachycardia in 8, repolarization abnormalities in 38, atrial tachycardia in 11, atrial fibrillation in 3, but there was constant persistence of sinus bradycardia in 1 adolescent and of nonspecific changes in ST-T segment in a preschooler.

Between 2008–2017 it was observed an increase of the prevalence of isolated atrial extrasystoles, isolated ventricular extrasystoles, incomplete right bundle branch block and sinus bradycardia, compared to 2007 (*p*<0.05). From 55 patients with repolarization abnormalities before the first antiparasitic treatment 30 (54.5%) were found with T-wave inversion, 3 (5.5%) with ST alteration and 22 (40%) with both T-wave inversion and ST alteration. All the 22 patients with nonspecific changes in ST-T segment between 2008–2017 had T-wave inversion, none had ST alterations. No patients had heart rate less than 40bpm. Some ECG/Holter abnormalities as low voltage, 2^nd^ AV block, left bundle branch block or increase of the QT interval were not found between 2008–2017 (**Table 2**). The frequency of atrial and ventricular extrasystoles was not higher than 10% of heart beats in any case. 1^st^ AV block was only described in all ECG/Holter performed between 2008–2017 in the same person (female, 65 years with a history of hypertension without ECG of 2007). The only patient with atrial fibrillation between 2012–2014 had arterial hypertension and hypothyroidism. One patient had non-sustained ventricular tachycardia in non-permanent manner in 2007, and 2002–2014. Atrial rhythm and abnormal union rhythm (data do not appear in **Table 2**) were reported in the Holter of the same patient (female, 7 years old) in the 2009–2011 evaluation, this finding was not found in subsequent evaluations; however, a 15 years-old boy had atrial rhythm inconsistently in two opportunities). No patients presented any electrocardiographic abnormality suggestive of CCC.

During follow up there was a higher risk of isolated atrial extrasystoles, isolated ventricular extrasystoles, incomplete right bundle branch block, sinus bradycardia and sinus tachycardia.

**Fig 3:**
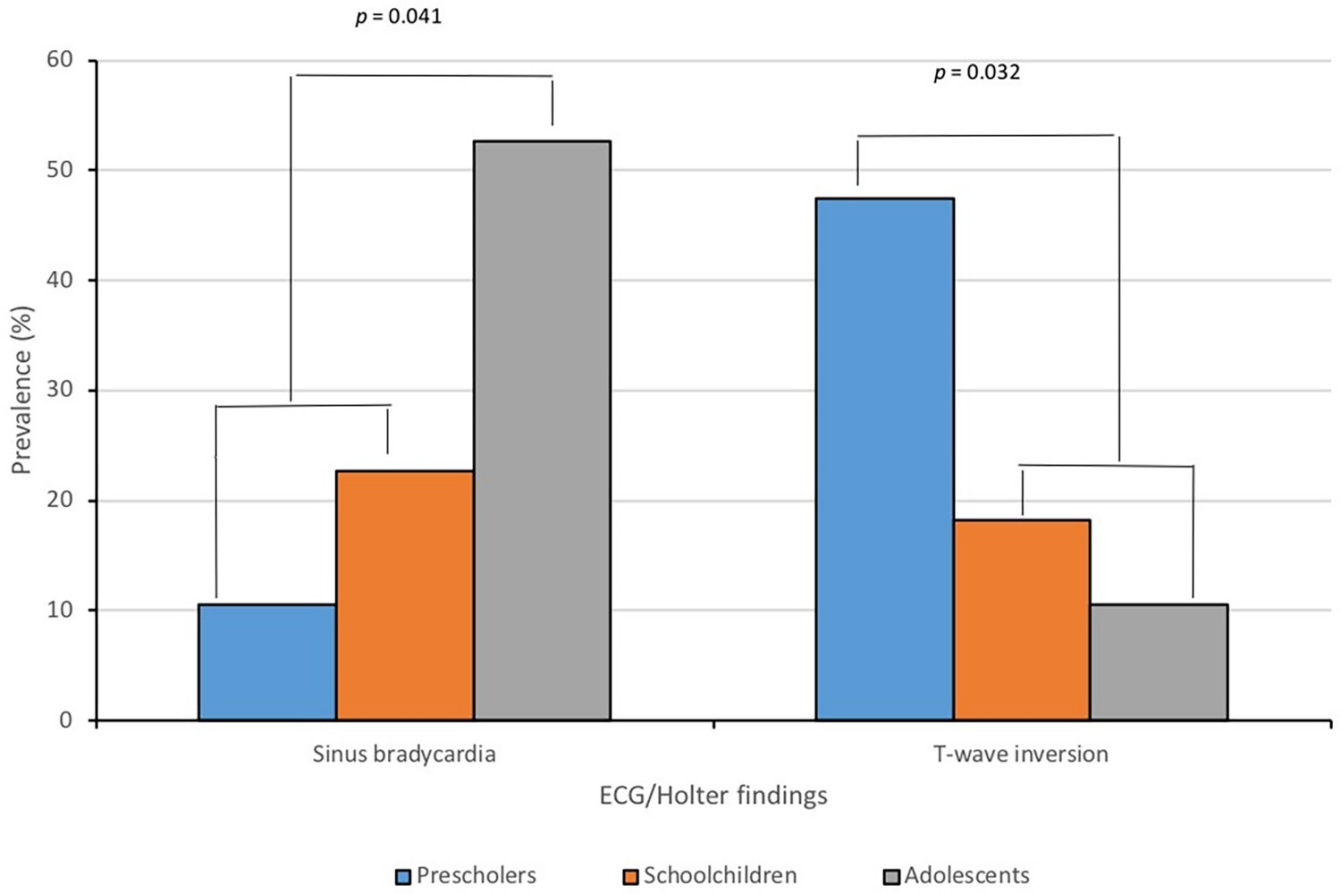
Post-treatment ECG/Holter findings with statistical significance according to age of children with oral Chagas disease, Chacao, Caracas, Venezuela, 2008–2017 (Abnormalities without statistical significance were not shown).

**Table 3** shows the values of the significant pre- and post-treatment according the age group ECG/Holter results. Between 2008–2017, there was not significant difference between ECG/Holter abnormalities prevalence and sex of the patients. However, the prevalence of some ECG/Holter findings had age-related differences. In the pre-treatment lapse atrial tachycardia, sinus tachycardia, ventricular tachycardia, atrial fibrillation, low voltage, isolated ventricular extrasystoles were more frequent in adults, and repolarization abnormalities (in the pre-treatment) and sinus bradycardia (during the post-treatment period) were more frequent in children. Regarding the age of the children, it was observed that repolarization abnormalities were more frequent among preschoolers and schoolchildren compared to adolescents, contrary to sinus bradycardia, which was significantly more frequent in adolescents (**Fig 3**). The OR and 95% CI values ​​only showed a probable causal association between adult age and the prevalence of atrial and ventricular tachycardia, sinus tachycardia, atrial fibrillation, low voltage in the year 2007 and isolated atrial extrasystoles, isolated ventricular extrasystoles in the period 2008–2017.

### Effect of the second treatment on the post-treatment electrocardiographic evaluation

The second treatment did not have a statistically significant influence on the incidence of ECG/Holter findings.

### Echocardiogram (ECHO)

It was not done systematically in each year or period (**Table 4**). 1.7 (range 0–8) ECHO’s were performed per person. During the acute phase of ChD in 2007, the finding of pericardial effusion with or without decreased ejection fraction and diffuse hypokinesia predominated since 10 of 16 patients (62.5%) had this finding. After the first antiparasitic treatment, the most reported findings in 90 ECHO performed were mild pericardial effusion in two persons (2.2%) and left ventricular hypertrophy in 5 (5.6%); both pericardial effusion and left ventricular hypertrophy were diagnosed only in adults over 50 years of age with history of hypertension. There was statistical difference when comparing pericardial effusion before and after treatment (*p* = 0.012 with OR 1.200 and 95%CI 0.839–1.716). The 2008–2017 post-treatment ECHO did not show left ventricular dysfunction in any patient, in fact, from 90 ECHOs performed after treatment 83 (92.3%) were normal in comparison with 2007; there was statistical difference (*p* = 0.022 with OR 0.091 and 95%CI 0.005–1.547)

**Table 4:** Altered echocardiographic findings and cardiothoracic ratio in the follow-up in patients with Chagas disease acquired by the oral route, Chacao, Caracas, Venezuela, 2007–2017.

Study	Findings	Prevalence of alterations for year or period (diagnose/evaluated)					
		Pre-treatment	Post-treatment				
		2007	2008	2009–2011	2012–2014	2015–2017	2008–2017
**Echocardiogram** *	Pericardial effusion	11/16 (68.8%)	1/11 (9.1%)	1/22 (4.5%)	1/82 (1,2%)	0/14 (0%)	2/90 (2.2%)**
	Ejection fraction <40%	3/16 (18.8%)	0/11 (0%)	0/22 (0%)	0/82 (0%)	0/14 (0%)	0/90 (0%)
	Diffuse hypokinesia	1/16 (6.3%)	1/11 (9.1%)	0/22 (0%)	0/82 (0%)	0/14 (0%)	1/90 (1.1%)
	Left ventricular hypertrophy	2/16 (12.5%)	0/11 (0%)	0/22 (0%)	4/82 (4.90%)	6/14(42.9%)	6/90 (6.7%)
**Chest-X -ray**	Cardiothoracic ratio > 0.5	2/5 (40%)	0/4 (0%)	0/4 (0%)	7/55 (12.7%)	9/87 (10.3%)	10/83 (10.8%)

*Some patients had more than one abnormality. Not considered in this table: Patent *foramen ovale* in two patients, mild mitral, tricuspid or pulmonary regurgitation in 3 patients, aortosclerosis in one patient, Inferior face aneurysm was reported in one study in 2012–2014, but was not corroborated in later evaluations

### Chest-X-ray

Each patient underwent 1.1 (0–3) chest X-rays during follow-up, in fact 93/106 underwent at least one chest-X-ray during the observation period. Of the 5 patients who underwent chest-X-ray in 2007, only one new X-ray was done in the period 2012–2014 and was normal in all of them. The 4 evaluated in 2008 are different from the 4 evaluated in 2009–2011 and 2007. During 2008–2017 there were 10 persons with CTR increased (**Table 4**) 7 were women (6 with some underlying pathology such as hypertension, dyslipidemia, or hypothyroidism) and 3 were children aged 5, 8, and 11, respectively.

The first appearance of some clinical or ECG/Holter findings was more frequent during the early post-treatment phase (incomplete right bundle branch block, sinus tachycardia, T-wave inversion), others as dizziness and clinical bradycardia were first found at the end of the observation period. Palpitations, fatigue, precordial pain, isolated atrial extrasystoles, isolated ventricular extrasystoles and sinus bradycardia first appeared more frequent in the middle phase. In 2008, a 61-years-old patient had atrial premature complex.

Between the first and the 8–10 years of observation, the incidence of dizziness and clinical bradycardia rising 1.9–7,8 times (9.5/4.9 and 14.8/1.9 respectively); less noticeable was the increased of the apparition of new cases of palpitations, chest pain, isolated ventricular extrasystoles and sinus bradycardia on ECG/Holter (**Table 5**). During the first and the 8–10 years after treatment, the incidence of some findings decreased (tiredness or fatigue and isolated atrial extrasystoles) or reached zero value (incomplete right bundle branch block, sinus tachycardia, T-wave inversion and atrial tachycardia) (**Table 5**).

**Table 5:** Cumulative incidence of clinical and ECG/Holter findings after treatment in 106 patients with Chagas disease orally acquired, Chacao, Caracas, Venezuela, 2008–2017.

Clinical and ECG/Holter abnormality evaluated		Cumulative incidence after the first treatment*	
		1 year	8–10 years
Clinical	Palpitations	9/103 (8.7%)	5/47 (10.6%)
	Tiredness or fatigue	17/103 (16.5%)	5/48 (10.4%)
	Chest pain	10/103 (9.7%)	6/53 (11.3%)
	Dizziness	5/103 (4.9%)	9/95 (9.5%)
	Bradycardia	2/103 (1.9%)	12/81 (14.8%)
ECG/Holter	Isolated atrial extrasystoles	4/102 (3.9%)	1/81 (1.2%)
	Isolated ventricular extrasystoles	1/105 (1.0%)	2/89 (2.2%)
	Incomplete right bundle branch block	18/102 (17.6%)	0/81 (0%)
	Sinus bradycardia	5/102 (4.9%)	4/85 (4.7%)
	Sinus tachycardia	16/102 (15.7%)	0/88 (0%)
	T-wave inversion	15/102 (14.7%)	0/84 (0%)
	Atrial tachycardia	2/102 (2.0%)	0/97 (0%)
	Non-sustained ventricular tachycardia	0/102 (0%)	0/105 (0%)
	Atrial fibrillation	0/102 (0%)	0/105 (0%)
	Long QT interval	0/102 (0%)	0/106 (0%)
	Low voltage	0/102 (0%)	0/106 (0%)

*Incidence calculation is shown in S1–S3 Data.

**Fig 4:**
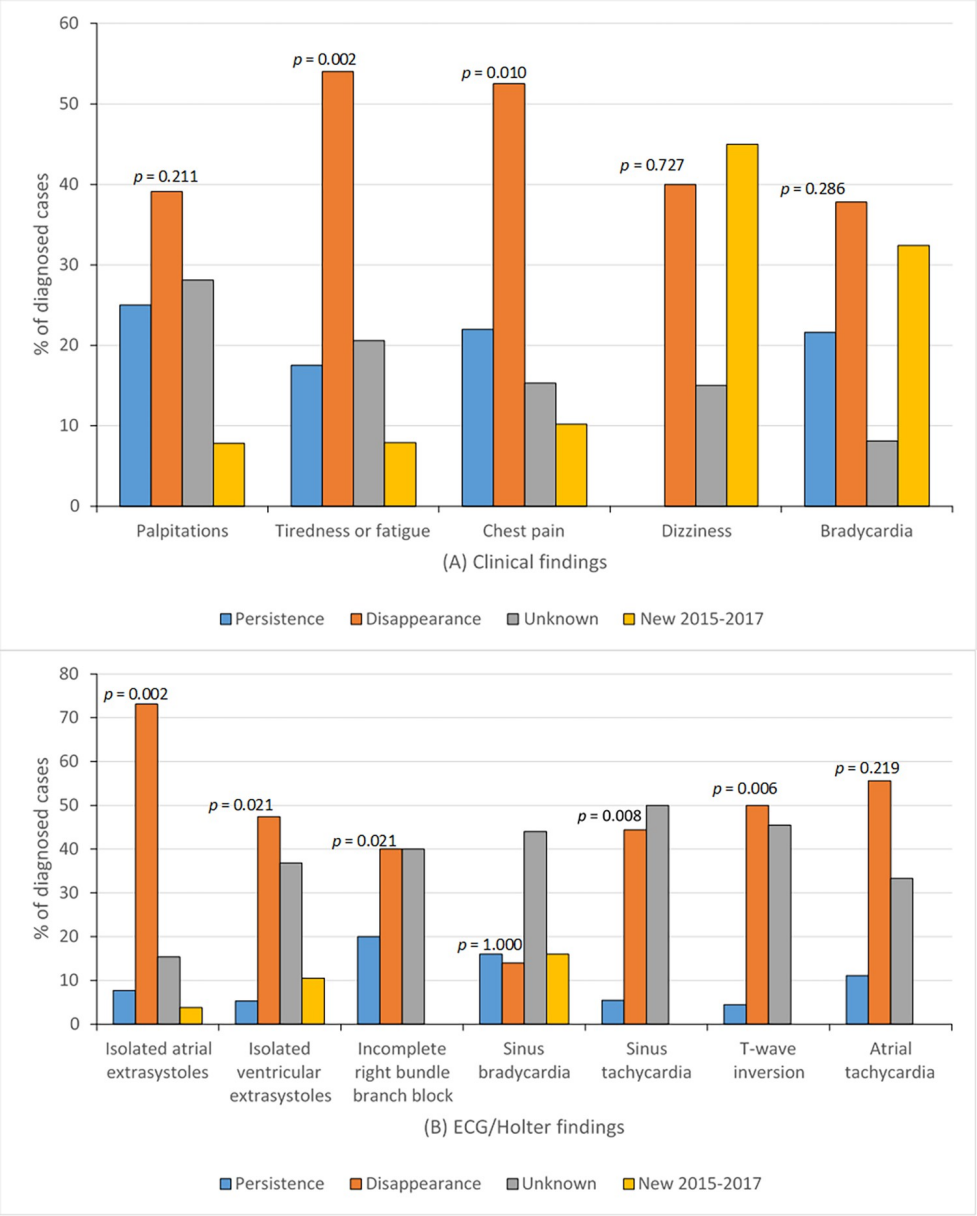
Definitive persistence or disappearance of clinical (A) or ECG/Holter (B) findings after the first treatment 2008–2017, in patients with Chagas disease acquired by the oral route, Chacao, Caracas, Venezuela (*p* value according to the McNemar χ^2^ test comparing persistence *versus* disappearance).

Incidence of definitive improvement or persistence of clinical or ECG findings are shown in **Fig 4**. This figure excludes those cases without prior information, as well as new cases diagnosed between 2015–2017 whose evolution is unknown. In all cases (except dizziness and sinus bradycardia) the definitive improvement of signs/symptoms and ECG/Holter findings exceed in percentage terms their constant persistence, however, there was statistically significant difference when comparing improvement and persistence of tiredness or fatigue, chest pain, isolated atrial extrasystoles, isolated ventricular extrasystoles, incomplete right bundle branch block, T-wave inversion and sinus tachycardia (**Fig 4**). Some persistent abnormalities were more frequent in children. From 8 cases with persistent clinical bradycardia, 6 (75%) were children, as well as all 4 persistent sinus bradycardia ones all cases of persistent incomplete right bundle branch block were found in 5 boys aged 5, 6, 8 and 14 years-old. The only case of T-wave inversion persistent was found in a 3-year-old preschooler. Other infrequent persistent abnormalities as isolated atrial extrasystoles, isolated ventricular extrasystoles, sinus tachycardia, atrial tachycardia and atrial fibrillation were found only in adults, some of them with another underlying pathology. In relation to the 24 patients who required hospitalization in the acute phase, among them 2 adults with underlying pathology, there was persistence of ECG lesions (isolated atrial or ventricular extrasystoles). There was persistence of one or several clinical manifestations that appeared from the year 2008 in 11 of the 24 hospitalized patients, of these, 7 were adults with underlying pathology, and 5 without (4 children and one adult). Of the persistent symptoms, the only one that we can objectively measure was clinical bradycardia that persisted in 2 adults with underlying pathology and 6 children. In no cases there was a statistically significant association between clinical or electrocardiographic parameter that improved definitely with the second treatment

## Discussion

Clinical and ECG follow-up data of 106 patients infected simultaneously with the same isolate TCI of *T*. *cruzi* [4,10] mostly treated with the same medication (NFX) [11] is analyzed. It would be expected that this group of patients evolve faster towards CCC compared to people with ChD acquired by other infection mechanisms due to the great clinical severity and the high percentage (86%) of ECG alterations evidenced during the acute phase [8]. Information is shown not only on the evolution of symptoms and ECG changes in the acute phase, but also whether there was any finding suggestive of CCC, information that is not explicit in other publications. In addition, data on the evolution in children and adults allows age comparisons and also criteria are proposed to define persistence/disappearance of the findings.

Unfortunately, we do not have all the ECG or ECHO of patients at the beginning in 2007 because some were seen in other health centers. As time progressed the population decreased as many migrated to other countries. However, a continuous and insistent effort was made to call patients to achieve a high percentage of patients evaluated until the end of the observation period in a country crossing a complex humanitarian crisis [26].

In this study we observed that the count of clinical or ECG abnormalities gives the false impression of an increase in the number or severity of findings. When we evaluated the dynamics of appearance, improvement or permanence of the findings, we had a better idea about what happened with this group of patients.

During follow-up none of our patients seem to have developed CCC, nor have there been deaths attributable to this disease. Even though this period appeared several symptoms and ECG findings different from those observed in the acute phase, none of them, appear to be attributable to CCC. However, it is important to take into consideration the clinical or ECG alterations that appeared or remained persistent since these events could be early manifestations of CCC, as sinus bradycardia.

Compared to Ortiz et al (2019) [27] who found that 33% of 63 patients presented ECG alterations during the acute phase of OChD, in the present study we found higher frequency (86%) and severity of cardiac lesions, however, already in the first post-treatment evaluations (2008), all serious lesions such as non-sustained ventricular tachycardia, atrial fibrillation, low voltage and 1^st^ or 2^nd^ AV block disappeared, including those of the 14-year-old boy who required cardioversion. In fact, few months after the acute phase was over, 50% of patients with abnormal ECGs were considered normal. Bastos et al (2010) [28] reported that after the third month of treatment, ECG readings normalized in 91.7% of patients who were infected during two micro-outbreaks of OChD in two northeastern Brazilian towns. There are reports of persons who did not improve, as a patient with acute OChD with atrial fibrillation who only improved 9 months after cardiac surgical ablation via a catheter and amiodarone [29]. Pinto et al (2010) [30] found higher frequency of post-treatment ECG alterations (3.9 years post-treatment) in patients who had shown ECG abnormalities during the acute phase in comparison with those who had not.

The fact that none of our patients have presented ECG with low voltage, anterosuperior divisional block, pathologic Q wave, right bundle branch block, or extrasystoles in more than 10% of heart beats during the follow-up period could be associated or related to a good prognosis for not developing CCC in the future, since all these findings are included in the diagnostic risk score developed by Brasil et al (2016) [31] to predict the appearance or development of CCC. The only patient with 1^st^ AV block has hypertension that could explain these anomalies [32]. Unfortunately, we do not know if this patient had these findings in 2007 because we do not have her ECG, but in 2008, these cardiac lesions were already described. Between 2012 and 2014 an aneurysm in the inferior face of the heart was reported not confirmed in further ECHOs in a patient with atrial fibrillation, extrasystoles and other abnormalities. We assume that it was miss-interpretation in a patient with a very pathological ECG.

Just as most of the serious or severe ECG findings of the acute phase disappeared or improved rapidly, the incidence of others (atrial arrhythmia, isolated ventricular extrasystoles increased in the period corresponding to the chronic phase, mainly in the last five years of observation. Incidence of incomplete right bundle branch block increased suddenly after treatment and then fell until it disappeared, as well as that of sinus tachycardia. Sinus bradycardia was an ECG finding whose incidence gradually increased over the years, and, unlike the other ECG abnormalities, its persistence prevailed over its total improvement.

In a 7-year follow-up study in Bahia (Brazil), Maguirre et al (1987) [33] found that most people acquired ChD before the age of 20 and that those with early right bundle branch block and other ventricular conduction defects (VCDs) had greater chance of developing additional ECG abnormalities or death compared to those who did not have these lesions early in life. These authors suggest that CCC develops early along with *T*. *cruzi* infection and that ECG during the early asymptomatic stage of infection was able to distinguish persons with potentially lethal cardiac lesions from those with a benign prognosis. In our study despite the rapid improvement of all the abnormalities in the first year after treatment, we found 5 children aged 5–14 years with persistent right bundle branch block through the follow up.

Our results are comparable to those of Neves et al (2020) [34], because they also did not find CCC at 10 years of observation in the large cohort on patients from different outbreaks of OChD in Brazil. But, these same authors, had reported that 3.9 years after treatment for acute OChD, in 11 people in Belen (Brazil), three patients presented ECG abnormalities consistent with chronic ChD [35]. Falk et al (2022) reported that patients with ECG findings consistent with acute myocarditis during the acute phase of ChD improved, and apparently none developed CCC at 10 years of observation [36]. Of this cohort, only 2 patients (who had a normal baseline ECG) developed one incomplete right bundle branch block and the other isolated ventricular extrasystoles [36]. In our casuistry, although we did not find any patients with CCC, the number of altered ECG findings during follow-up is higher.

During the acute phase of ChD, the most frequent ECG finding found was repolarization abnormalities in 64%, a fairly high percentage compared to 13% reported by Ortiz et al (2019) [27], however, its incidence as well as that of atrial tachycardia, decreased progressively until it was no longer diagnosed. It is likely that these findings are associated with the acute phase, as reported in various OChD microepidemics. Mendoza et al (2011) described that atrial tachyarrhythmias were frequent manifestations of acute OChD [37]. Ventricular repolarization abnormalities persisted in 50% of the patients at 180 days of follow-up [28], compared to 19% prevalence in our patients in the first year of observation.

Age seems to play an important role in explaining the higher frequency of some findings. From a clinical point of view, it is possible that the palpitations, fatigue/tiredness and precordial pain mentioned more frequently by adults, can be explained because children do not know how to report some symptoms. In relation to the ECG/Holter, our results allow us to infer that being an adult was a risk factor for presenting some alterations such as sinus tachycardia, atrial and ventricular tachycardia, atrial fibrillation, and low voltage. Despite the fact that children were the most affected in the acute phase, the ECG lesions were more severe in adults. After treatment, atrial arrhythmia and isolated ventricular extrasystoles predominated in adults perhaps associated with underlying pathologies such as arterial hypertension. In children, the finding of repolarization abnormalities predominated in the acute phase of ChD. After the first treatment, this finding was more common among preschoolers; in the chronic phase of ChD, sinus bradycardia was diagnosed more frequently in children (especially adolescents) compared to adults, and, although both ECG findings can be considered as non-pathological in these age groups due to practice sports activities or physical training, it is important to do a thorough cardiological follow-up, especially in those cases with persistent repolarization abnormalities or sinus bradycardia in order to rule out CCC. Age of children at the acute phase does not seem a determining aspect in the evolution of ChD.

Despite the fact that some clinical (palpitations) findings were more frequent among women after treatment, in this cohort of patients we were unable to demonstrate a causal association between gender and cardiological alterations unlike what was reported in the Regional Agreement of the Experts on Chagas of the South American Cardiology Societies [38] and what was found in other infectious pathologies that affect the heart, such as Zika, which can cause severe myocarditis, heart failure, serious arrhythmias more frequent in women [39]. We did not find any difference related to the gender al ECG abnormalities after treatment.

Regarding subjective clinical data, palpitations have a different meaning in the acute or chronic phase. In the acute phase, it is associated with fever and stress in patients informed of the risk of presenting CCC in the future. In the chronic phase, palpitations could be the expression of extrasystoles. Fatigue/tiredness, precordial pain and dizziness were reported frequently only after the acute phase of ChD had passed. Regarding fainting/syncope reported by two patients, it could be explained by other causes not inherent to CCC. Even when isolated subjective clinical data do not seem to be manifestations of CCC at 10 years of evaluation, it is important to assess whether they last over time in people who eventually develop CCC. In relation to objective clinical findings such as clinical bradycardia, its incidence gradually increased as the observation period progressed. It is unlikely that this is associated with rhythm disorders or blockages, but with physical activity, especially among young people, as we pointed out with sinus bradycardia.

The severity of the clinical manifestations of the acute phase (determined according to the need for hospitalization) [8] observed in 22.6% of the 106 patients, does not seem to have influenced or determined the appearance and persistence of the findings since no association with hospitalization was found.

In a large cohort of 199 children and 90 adults treated with NFX for acute ChD (congenital, undetermined, vectorial or transfusion), almost 90% asymptomatic collected in 10 years, the clinical manifestations of acute phase of ChD improved although the timing of the improvement in that observation period is unclear [36]. In our study the improvement or disappearance of pathological clinical findings was observed, few months after antiparasitic treatment. In all cases, the incidence of clinical improvement was higher than the incidence of persistence of pathological clinical findings.

ECHO, Doppler and magnetic resonance-imaging (MRI) techniques provide useful structural and functional information in the detection of early myocardial damage, risk assessment of prognosis, disease progression, and management of patients with ChD [40]. During the acute phase Ortiz et al (2019) [27] reported pericardial effusion in 10% of cases, in our patients 68.8% presented this finding. It is possible that we are overestimating the true prevalence of pericardial effusion considering the small number of ECHOs available, and it is also likely that this study was performed in the most severe patients. As well as Ortiz et al (2019) [27], we observed that all the serious alterations found in the ECHO during the acute phase of ChD improved early after treatment. The systolic ejection fraction normalized in two out of three patients, while pericardial effusion disappeared after 180 days [28]. In our case, the 3 patients who had EF<40% improved within a few weeks. The finding of LVH in the post-treatment ECHO was associated with the presence of hypertension [41].

The results of the chest-X-ray do not allow us to infer whether the increase in CTR is due to CCC or to other pathologies, however, the finding of CTR >0.5 in 3 children is a fact that should draw attention, because they had two studies in different years showing CTR >0,5. We do not know if the few chest-X-rays performed during the acute phase of ChD and reported as normal 5–7 years later, were normalized at this stage or before, since we do not have previous radiological information. The chest-X-ray is a study, generally within the reach of the clinician, and it is useful in patients with some underlying pathology such as hypertension. However, its findings may be subjective depending on the quality of the image and the homogeneity in the criteria for reporting the results of CTR, on the other hand, the value of CTR is not always correct and increases the number of false-positive results, especially in obese or older subjects who may have a misdiagnosis of cardiac enlargement [25] and there may be variations depending on the age and sex of the patient [42].

The persistence of signs/symptoms or ECG abnormalities that appeared once the acute phase of ChD was over as a risk factor for the development of CCC in the future should be evaluated. It would have been excellent to be able to recommend, that every patient with *T*. *cruzi* infection with palpitations or persistent fatigue or sinus bradycardia has a percentage risk of developing CCC, but our results of 10 years follow-up, do not allow us to make these affirmations. The persistence of right bundle branch block in five children draws attention to this ECG finding, which should be monitored frequently.

In patients who became infected in different outbreaks of acute ChD including oral, vectorial and other transmission, in Pará, Amazonas and Amapá (Brazil) since 1996, Neves et al (2020) [34], carried out a descriptive cohort study in which they evaluated for 10.9 years 126 children and adolescents. The majority of patients with cardiac involvement in the acute phase of ChD evolved favorably, but 2.4% (3/126) persisted with cardiac injury until the end of the observation period (nonspecific changes in ST-T segment in 28) [34]. In the present study, two children presented sinus bradycardia and one ventricular repolarization abnormalities since the acute phase up to present day, but we do not know if these persistent findings mean these two children will have greater chance of developing CCC in the future. Each of these findings by itself may not be abnormal. However, it is necessary to carry out a strict cardiological follow-up in these patients. We do not know if the higher percentage of persistence of some lesions observed by Neves et al (2020) [34] is related to different types of acute phase, since they include children who were infected by the oral route and others by the vectorial route.

When we evaluated this population for the first time, four weeks had already passed after the probable day of infection [10], the early and massive diagnosis of the entire population at risk, with the immediate administration of anti-parasitic treatment supervised for 3 months, prevented other deaths in addition to the one that occurred during the outbreak [10], it is possible that early diagnosis and treatment was responsible for avoiding mortality and morbidity. In another large outbreak of OChD, also school-based in Venezuela, the treatment intervention was delayed and consequently, morbidity and mortality were higher [43].

CCC probably will develop in the group of patients that remains infected (non-responders). It is likely that the administration of a second treatment to 48 people modified the natural course of the infection in an infected and treated population, preventing the early appearance of symptoms. The present study was not designed to demonstrate this point, however, other works suggest that the persistence of the parasite is associated with the pathology [44] and it is likely that successive anti-parasitic treatments, would reduce the parasite load, prevent greater cardiac damage.

The UN Refugee Agency (UNHCR) estimates that approximately 7 million people have migrated from Venezuela due to the serious social, political and economic crisis generated by violence, insecurity, threats, lack of food, medicine and essential services, becoming in the second largest external displacement crisis in the world [45]. This helps to explain the reduction in the population studied. Among these patients there are people with evidence of *T*. *cruzi* infection [12] becoming a probable source of man-man infection in other latitudes [6]

### Final remarks

Clinical and laboratory follow-up were undergoing in 106 *T*. *cruzi* orally infected patients in 2007. Based on laboratory tests patients were discriminated into responders (R) and non-responders (NR) to antiparasitic treatment. The same did not occur with clinical follow-up. We are preparing a work of integration of clinical and laboratory to associate the cardiac behavior with the R and NR condition.

The clinical protocol in the management of outbreaks of oral infection by *T*. *cruzi* should contemplate a thorough clinical evaluation including chest-X-ray, ECG and ECHO at the time of infection regardless morbidity. Subsequently, follow-up should be annual and include clinical evaluation, ECG and ECHO, and chest-X-ray every three years. In parallel, the evaluation of infection and morbidity markers.

In 10 years, CCC was not established in the group of patients evaluated. However, some ECG changes persisted such as sinus bradycardia, incomplete right bundle branch block or persistent T-wave inversion as well as left ventricular hypertrophy in patients with ChD and hypertension, and others with more erratic appearance, such as isolated atrial or ventricular extrasystoles or sinus tachycardia, atrial tachycardia, they will deserve special attention. There are not previous studies of successive anti-parasitic treatments in OChD, our work team proposes successive treatment as a point of discussion and considers that as long as there is evidence of parasitic persistence and there are not contraindications to treatment, it should be repeated in the young population every 5 years in order to avoid heart damage by direct action of the parasite

Since the ECG findings in the present study are not characteristic of CCC, we can classify all these patients under the indeterminate form of the ChD [46]. However, based on a systematic review and meta-analysis, published in 2020 [47], patients with the indeterminate form of ChD, have a significant progressive annual risk increase of develop CCC; this risk augment more than double in patients diagnosed with acute ChD [47]. For this reason, annual cardiology evaluation is essential, especially in those with persistent abnormalities.

The objective of this work was to detect not only declared CCC, but to detect early cardiologic findings that could predict progression towards CCC, hence, evaluating persistence of symptoms or ECG findings has seemed very important to us.

In the case of ChD, the real magnitude or dimension of the problem in Venezuela is unknown since there is no official information on cases or prevalence in endemic areas, but, based on the data published by researchers from autonomous universities [46,47] and the growing number of reports of ChD acquired by the oral route [48–55], we estimate that the universe of infected *T*. *cruzi* population has increased.

## Supporting information

S1 Data Using palpitations as an example to calculate incidence after treatment of clinical or electrocardiographical findings in 106 patients with Chagas disease acquired by the oral route, Chacao, Caracas, Venezuela, 2008–2017. (TIF)

S2 Data Incidence of clinical findings in the follow-up of 106 patients with oral Chagas disease, Chacao. Caracas. Venezuela, 2007–2017.
(DOCX)

S3 Data Incidence of ECG/Holter abnormalities in 106 patients with Chagas disease orally acquired, Chacao, Caracas, Venezuela, 2007–2017. (DOCX)

S4 Data Incidence of clinical (A) and ECG/Holter (B) findings after treatment in 106 patients with Chagas disease orally acquired, Chacao, Caracas, Venezuela, 2008–2017.
(TIF)

S5 Data Definitive persistence or disappearance of clinical (A) or ECG/Holter (B) after the first treatment, 2008–2017, in patients with Chagas disease acquired by the oral route, Chacao, Caracas, Venezuela.
(DOCX)
