# Supplementary figures and images for: Ten years follow-up of the largest oral Chagas disease outbreak: Cardiological prospective cohort study
Source: PLoS Negl Trop Dis. 2023 Oct 6;17(10):e0011643. doi: 10.1371/journal.pntd.0011643 (PMC10584157; doi:10.1371/journal.pntd.0011643)

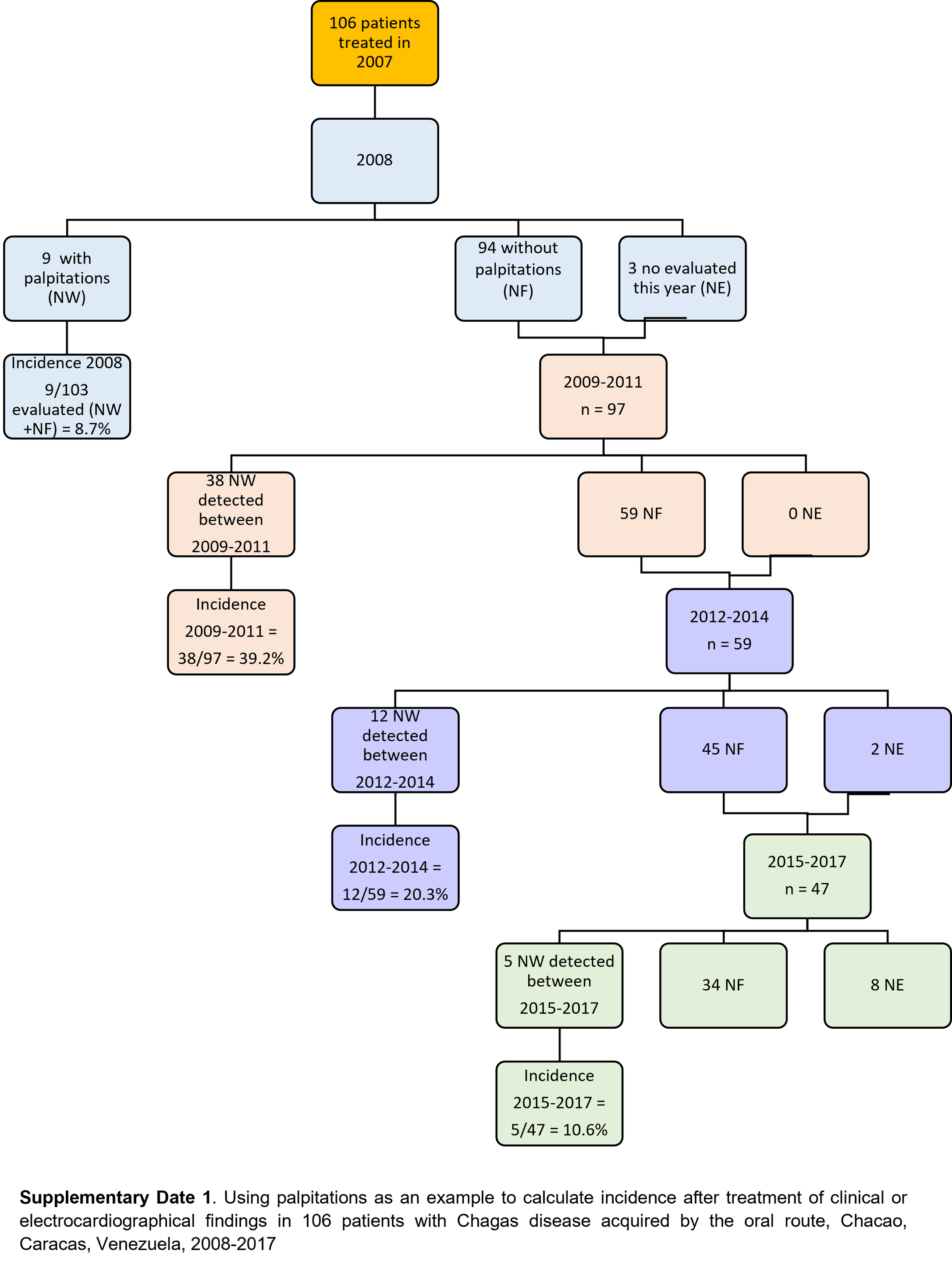

Supplement: S1 Data — (TIF) [file pntd.0011643.s001.tif]

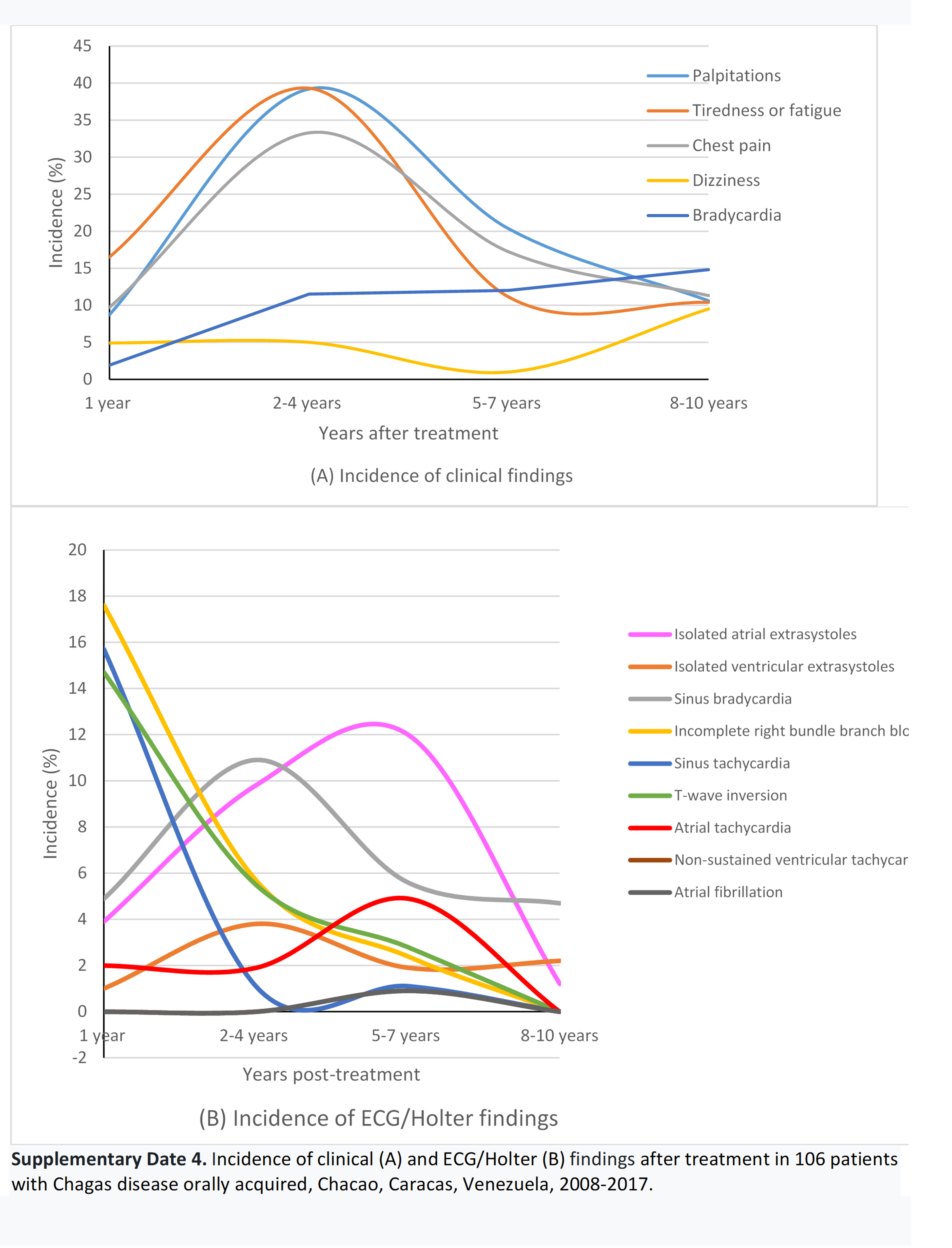

Supplement: S4 Data — Incidence of clinical (A) and ECG/Holter (B) findings after treatment in 106 patients with Chagas disease orally acquired, Chacao, Caracas, Venezuela, 2008–2017. (TIF) [file pntd.0011643.s004.tif]
