# Supplementary material for: Ten years follow-up of the largest oral Chagas disease outbreak: Cardiological prospective cohort study
Source: PLoS Negl Trop Dis. 2023 Oct 6;17(10):e0011643. doi: 10.1371/journal.pntd.0011643 (PMC10584157; doi:10.1371/journal.pntd.0011643)
Supplement: S2 Data — Venezuela, 2007–2017. (DOCX) [file pntd.0011643.s002.docx]

**Supplementary Date 2.** Incidence of clinical findings in the follow-up of 106 patients with oral Chagas disease, Chacao. Caracas. Venezuela, 2007-2017.

| Clinical finding | Year or period after the first treatment | Elements used to calculate the incidence* | | | | | |
| --- | --- | --- | --- | --- | --- | --- | --- |
|  |  | **New cases (NW)** | **Abnormality no found (NF)** | **Patient no evaluated (NE)** | **NF + NE** | **NW/NF + NE** | **Incidence** |
| Palpitations | **2008**** | **9** | 94 | 3 | 97 | 9/103** | 8.7% |
|  | **2009-2011** | **38** | 59 | 0 | 59 | 38/97 | 39.2% |
|  | **2012-2014** | **12** | 45 | 2 | 47 | 12/59 | 20.3% |
|  | **2015-2017** | **5** | 34 | 8 | 42 | 5/47 | 10.6% |
| Tiredness or fatigue | **2008**** | **17** | 86 | 3 | 89 | 17/103** | 16.5% |
|  | **2009-2011** | **35** | 54 | 0 | 54 | 35/89 | 39.3% |
|  | **2012-2014** | **6** | 46 | 2 | 48 | 6/54 | 11.1% |
|  | **2015-2017** | **5** | 31 | 12 | 43 | 5/48 | 10.4% |
| Chest pain | **2008**** | **10** | 93 | 3 | 96 | 10/103** | 9.7% |
|  | **2009-2011** | **32** | 64 | 0 | 64 | 32/96 | 33.3% |
|  | **2012-2014** | **11** | 51 | 2 | 53 | 11/64 | 17.2% |
|  | **2015-2017** | **6** | 37 | 10 | 47 | 6/53 | 11.3% |
| Dizziness | **2008**** | **5** | 98 | 3 | 101 | 5/103** | 4.9% |
|  | **2009-2011** | **5** | 96 | 0 | 96 | 5/101 | 5.0% |
|  | **2012-2014** | **1** | 91 | 4 | 95 | 1/96 | 1-0% |
|  | **2015-2017** | **9** | 70 | 16 | 86 | 9/95 | 9.5% |
| Bradycardia | **2008**** | **2** | 101 | 3 | 104 | 2/103** | 1.9% |
|  | **2009-2011** | **12** | 92 | 0 | 92 | 12/104 | 11.5% |
|  | **2012-2014** | **11** | 78 | 3 | 81 | 11/92 | 12.0% |
|  | **2015-2017** | **12** | 54 | 15 | 69 | 12/81 | 14.8% |
| Fainting? Syncope? | **2008**** | **0** | 103 | 103 | 3 | 0/103** | 0% |
|  | **2009-2011** | **1** | 105 | 0 | 0 | 1/103 | 0.9% |
|  | **2012-2014** | **0** | 101 | 4 | 105 | 0/105 | 0% |
|  | **2015-2017** | **0** | 88 | 17 | 105 | 0/105 | 0% |

* To calculate the incidence after 2008, we divided the number of new cases from those diagnosed these period by the sum of clinical alteration not found plus patients not evaluated the previous period.

**Evaluated in 2008 = 103
