# Supplementary material for: Ten years follow-up of the largest oral Chagas disease outbreak: Cardiological prospective cohort study
Source: PLoS Negl Trop Dis. 2023 Oct 6;17(10):e0011643. doi: 10.1371/journal.pntd.0011643 (PMC10584157; doi:10.1371/journal.pntd.0011643)
Supplement: S3 Data — (DOCX) [file pntd.0011643.s003.docx]

**Supplementary Date 3**. Incidence of ECG/Holter abnormalities in 106 patients with Chagas disease orally acquired, Chacao, Caracas, Venezuela, 2007-2017.

| Abnormality of ECG/Holter | Year or period after the first treatment | Elements used to calculate the incidence* | | | | | |
| --- | --- | --- | --- | --- | --- | --- | --- |
|  |  | **New cases (NW)** | **Abnormality no found (NF)** | **Patient no evaluated (NE)** | **NF + NE** | **NW/NF + NE** | **Incidence** |
| Isolated atrial extrasystole | **2008**** | 4 | 98 | 4 | 102 | 4/102** | 3,9% |
|  | **2009-2011** | 10 | 90 | 2 | 92 | 10/102 | 9.8% |
|  | **2012-2014** | 11 | 69 | 12 | 81 | 11/92 | 12.0% |
|  | **2015-2017** | 1 | 48 | 32 | 80 | 1/81 | 1.2% |
| Isolated ventricular extrasystole | **2008**** | 1 | 101 | 4 | 105 | 1/105** | 1.0% |
|  | **2009-2011** | 4 | 99 | 2 | 101 | 4/105 | 3.8% |
|  | **2012-2014** | 12 | 77 | 12 | 89 | 12/101 | 11.9% |
|  | **2015-2017** | 2 | 59 | 28 | 89 | 2/89 | 2.2% |
| Incomplete right bundle branch block | **2008**** | 18 | 84 | 4 | 88 | 18/102** | 17.6% |
|  | **2009-2011** | 5 | 82 | 1 | 83 | 5/88 | 5.7% |
|  | **2012-2014** | 2 | 70 | 11 | 81 | 2/83 | 2.4% |
|  | **2015-2017** | 0 | 59 | 22 | 81 | 0/81 | 0% |
| Sinus bradycardia | **2008**** | 5 | 97 | 4 | 101 | 5/102** | 4.9% |
|  | **2009-2011** | 11 | 89 | 1 | 90 | 11/101 | 10.9% |
|  | **2012-2014** | 5 | 73 | 12 | 85 | 5/90 | 5.6% |
|  | **2015-2017** | 4 | 53 | 28 | 81 | 5/85 | 4.7% |
| Sinus tachycardia | **2008**** | 16 | 86 | 4 | 90 | 16/102** | 15.7% |
|  | **2009-2011** | 1 | 88 | 1 | 89 | 1/90 | 1.1% |
|  | **2012-2014** | 1 | 76 | 12 | 88 | 1/89 | 1.1% |
|  | **2015-2017** | 0 | 62 | 26 | 88 | 0/88 | 0% |
| T-wave inversion | **2008**** | 15 | 87 | 4 | 91 | 15/102** | 14.7% |
|  | **2009-2011** | 5 | 84 | 2 | 86 | 5/91 | 5.5% |
|  | **2012-2014** | 2 | 74 | 10 | 84 | 2/86 | 2.3% |
|  | **2015-2017** | 0 | 56 | 28 | 84 | 0/84 | 0% |
| Atrial tachycardia | **2008**** | 2 | 100 | 4 | 104 | 2/102** | 2.0% |
|  | **2009-2011** | 2 | 100 | 2 | 102 | 2/104 | 1.9% |
|  | **2012-2014** | 5 | 84 | 13 | 97 | 5/102 | 4.9% |
|  | **2015-2017** | 0 | 64 | 33 | 97 | 0/97 | 0% |
| Non-sustained ventricular tachycardia | **2008**** | 0 | 102 | 4 | 106 | 0/102** | 0% |
|  | **2009-2011** | 0 | 104 | 2 | 106 | 0/106 | 0% |
|  | **2012-2014** | 1 | 92 | 13 | 105 | 1/106 | 0.9% |
|  | **2015-2017** | 0 | 70 | 35 | 105 | 0/105 | 0% |
| Atrial fibrillation | **2008**** | 0 | 102 | 4 | 106 | 0/102 | 0% |
|  | **2009-2011** | 0 | 104 | 2 | 106 | 0/106 | 0% |
|  | **2012-2014** | 1 | 92 | 13 | 105 | 1/106 | 0.9% |
|  | **2015-2017** | 0 | 70 | 35 | 105 | 0/105 | 0% |
| Increased QT interval | **2008**** | 0 | 102 | 4 | 106 | 0/102** | 0% |
|  | **2009-2011** | 0 | 104 | 2 | 106 | 0/106 | 0% |
|  | **2012-2014** | 0 | 93 | 13 | 106 | 0/106 | 0% |
|  | **2015-2017** | 0 | 71 | 35 | 106 | 0/106 | 0% |
| Low voltage | **2008**** | 0 | 102 | 4 | 106 | 0/102** | 0% |
|  | **2009-2011** | 0 | 104 | 2 | 106 | 0/106 | 0% |
|  | **2012-2014** | 0 | 93 | 13 | 106 | 0/106 | 0% |
|  | **2015-2017** | 0 | 71 | 35 | 106 | 0/106 | 0% |

* To calculate the incidence after 2008, we divided the number of new cases from those diagnosed these period by the sum of clinical alteration not found plus patients not evaluated the previous period.

**
