# Supplementary material for: Ten years follow-up of the largest oral Chagas disease outbreak: Cardiological prospective cohort study
Source: PLoS Negl Trop Dis. 2023 Oct 6;17(10):e0011643. doi: 10.1371/journal.pntd.0011643 (PMC10584157; doi:10.1371/journal.pntd.0011643)
Supplement: S5 Data — Definitive persistence or disappearance of clinical (A) or ECG/Holter (B) after the first treatment, 2008–2017, in patients with Chagas disease acquired by the oral route, Chacao, Caracas, Venezuela. (DOCX) [file pntd.0011643.s005.docx]

**Supplementary Date 5**. Definitive persistence or disappearance of clinical (A) or ECG/Holter (B) after the first treatment, 2008-2017, in patients with Chagas disease acquired by the oral route, Chacao, Caracas, Venezuela.

| **Clinical and ECG/Holter findings** | | **Diagnosed** | **Evolution (% of diagnosed)** | | | | ***p**** |
| --- | --- | --- | --- | --- | --- | --- | --- |
|  |  |  | **Persistence** | **Disappearance** | **Unknown** | **New 2015-2017** |  |
| Clinical | Palpitations | 64 | 16 (25%) | 25 (39.1%) | 18 (28.1%) | 5 (7.8%) | 0.211 |
|  | Tiredness or fatigue | 63 | 11 (17.5%) | 34 (54%) | 13 (20.6%) | 5 (7.9%) | **0.002** |
|  | Chest pain | 59 | 13 (22%) | 31 (52.5%) | 9 (15.3%) | 6 (10.2%) | **0.010** |
|  | Dizziness | 20 | 0 (0%) | 8 (40%) | 3 (15%) | 9 (45%) | 0.727 |
|  | Bradycardia | 37 | 8 (21.6%) | 14 (37.8%) | 3 (8.1%) | 12 (32.4%) | 0.286 |
| ECG/Holtr | Isolated atrial extrasystole | 26 | 2 (7.7%) | 19 (73.1%) | 4 (15.4%) | 1 (3.8%) | **0.002** |
|  | Isolated ventricular extrasystole | 19 | 1 (5.3%) | 9 (47.4%) | 7 (36.8%) | 2 (10.5%) | **0.021** |
|  | Incomplete right bundle branch block | 25 | 5 (20%) | 10 (40%) | 10 (40%) | 0 (0%) | **0.021** |
|  | Sinus bradycardia | 25 | 4 (16%) | 6 (14%) | 11 (44%) | 4 (16%) | 1.000 |
|  | Sinus tachycardia | 18 | 1 (5.5%) | 8 (44.4%) | 9 (50%) | 0 (0%) | **0.008** |
|  | T-wave inversion | 22 | 1 (4.5%) | 11 (50%) | 10 (45.5%) | 0 (0%) | **0.006** |
|  | Atrial tachycardia | 9 | 1 (11.1%) | 5 (55.6%) | 3 (33.3%) | 0 (0%) | 0.219 |
|  | Non-sustained ventricular tachycardia | 1 | 0 (0%) | 1 (100%) | 0 (0%) | 0 (0%) | … |
|  | Atrial fibrillation | 1 | 1 (100%) | 0 (0%) | 0 (0%) | 0 (0%) | … |
|  | Increased QT interval | 0 | 0 (0%) | 0 (0%) | 0 (0%) | 0 (0%) | … |
|  | Low voltage | 0 | 0 (0%) | 0 (0%) | 0 (0%) | 0 (0%) | … |

**p* value according to the McNemar χ^2^ test comparing persistence *versus* disappearance
